# Adulteration and quality assessment of tomato paste: a study of the Lebanese market

**DOI:** 10.3389/fnut.2025.1559287

**Published:** 2025-04-28

**Authors:** Fatima Habib, Salma Khazaal, Elie Bou Yazbeck, Espérance Debs, Suhair Sunoqrot, Nicolas Louka, Nada El Darra

**Affiliations:** ^1^Department of Nutrition and Dietetics, Faculty of Health Sciences, Beirut Arab University, Beirut, Lebanon; ^2^Department of Nutrition and Food Sciences, Faculty of Arts and Sciences, Holy Spirit University of Kaslik, Jounieh, Lebanon; ^3^Ministry of Economy and Trade, Beirut, Lebanon; ^4^Department of Biology, Faculty of Arts and Sciences, University of Balamand, Tripoli, Lebanon; ^5^Department of Pharmacy, Faculty of Pharmacy, Al-Zaytoonah University of Jordan, Amman, Jordan; ^6^Unité de Recherche Technologies et Valorisation Agro-Alimentaire, Centre d’Analyses et de Recherche, Faculté des Sciences, Université Saint-Joseph de Beyrouth, Beirut, Lebanon

**Keywords:** Tomato paste, food adulteration, quality parameters, starch usage, total soluble solids, Bostwick consistency, titratable acidity, color

## Abstract

Food adulteration has emerged as a significant global issue, impacting consumer health and fair-trade practices. This study aimed to evaluate the quality and potential adulteration with starch in tomato paste products available in the Lebanese market. A total of 41 local and imported tomato paste samples, without starch declarations, were collected from the Lebanese market and analyzed for starch usage and various quality parameters (total soluble solids, Bostwick consistency, viscosity, titratable acidity, color, and dry matter content), as well as compliance with Libnor and Codex Alimentarius standards. Results revealed that 37% of samples failed to meet starch usage standards, and 27% did not comply with the required total soluble solids (>24%), while all samples complied with acidity standards (<7%). Compliant samples had significantly higher values for total soluble solids, acidity, dry matter, and color compared to non-compliant ones (*p* < 0.01). A comparison of local and imported tomato paste products showed no significant differences in physicochemical properties, color, shelf life, or price, with parameters being similar across samples. Among local samples, 48% did not comply with the starch usage standard, and 26% failed to meet the required total soluble solids level. In contrast, imported samples adhered to starch usage standards, although 30% did not comply with TSS levels. This study highlights the prevalence of adulteration in local and imported tomato paste products in Lebanon and calls for further enforcement measures to ensure consumer protection and fair trade.

## 1 Introduction

Tomatoes are one of the most widely grown and significant crops in the world (1). In 2022, they emerged as the most-produced vegetable, with a total production of 186 million tons (2). Tomato-based products like juice, sauces, and ketchup are in high demand worldwide (3), with approximately 75% of them being concentrated into a paste (4, 5). This concentrated paste is typically stored for up to 2 years and either sold in its concentrated form or further diluted to create value-added products like sauces, salsas, and ketchup (5–7). Tomato paste is a primary component of tomato products, making it crucial to monitor and preserve its quality throughout the production process (7).

Local and international regulations establish the minimum quality standards and allowable ingredients for tomato processing, aiming to protect consumer health and promote fair practices in the food trade (8). The Codex Alimentarius defines “processed tomato concentrate” as a product that concentrates the juice or pulp of ripe, healthy red tomatoes (*Lycopersicon/Lycopersicum esculentum*). This concentrate undergoes straining or similar processes to eliminate most skins, seeds, and other coarse materials, resulting in a smooth final product preserved using physical methods. Besides tomato fruit, the ingredients allowed during processing include salt, spices, aromatic herbs, their natural extracts, lemon juice, and water (9).

Food adulteration refers to the practice of compromising food quality by incorporating foreign substances or removing essential components to increase profit margins (10, 11). This can occur in two forms: intentional adulteration, which involves deliberate actions by producers, and accidental adulteration, resulting from non-compliance with proper production practices (10). The intentional addition of foreign materials or ingredients is the most prevalent type of adulteration in processed foods. These practices degrade the overall quality of food and, in some cases, can pose serious health risks to consumers (12, 13). Additionally, certain non-food substances are deliberately introduced into food products to enhance their appearance or modify their characteristics.

In the European Union, the use of additives in concentrates is subject to strict regulations. These regulations specify the allowable limits for acidity regulators and salt in the final product. Along with the prohibition of colorants (14), it is necessary for all ingredients involved in the product’s manufacture to be listed on the packaging label (15). Consequently, the use of unauthorized external ingredients is considered unethical due to the associated health risks and the manipulation of consumers for unfair economic gain (16).

Despite existing regulations, incidents of food fraud are increasing, often driven by the desire to cut production costs or enhance product appeal for greater profits. Identifying food fraud is difficult because it is not easily detectable by consumers, and those involved often use sophisticated methods to evade detection (17). Besides, the risks associated with economically motivated food adulteration (EMA) may be greater than those posed by traditional food safety hazards, as the contaminants involved are frequently unknown and unconventional (18).

Although globally popular and highly susceptible to adulteration, commercial tomato paste has received limited research attention. Standard quality control practices for tomato paste production include hourly testing of freshly produced samples from each production line, evaluating parameters such as soluble solids, viscosity, consistency, pH, acidity, and color (7, 19). Its classification is primarily based on additive content and total soluble solids (TSS) levels (20). According to the Codex Alimentarius (21), tomato paste must contain at least 24% TSS and be free of additives (21). Key quality indicators, such as Bostwick consistency and viscosity, are critical in determining consumer acceptability and form an integral part of the product’s quality grade standards. In addition to their importance for the final product’s quality and consumer preference, consistency and viscosity also have significant economic implications for the tomato industry. Higher consistency and viscosity in processed tomatoes help reduce production costs by decreasing the amount of raw tomatoes required to achieve a certain product quality level (22). Titratable acidity (TA) is another crucial parameter, influencing both the safety and flavor of tomato paste. Acidity levels are affected by various factors, including the tomato cultivar, ripeness at harvest, processing method (hot vs. cold break), growing location, and seasonal variations (4, 22, 23). Citric acid, the primary acid in tomatoes, is the main contributor to the overall titratable acidity (24). As for color, it is a key quality attribute that greatly affects consumer acceptance. Several reactions can alter the product’s color during thermal processing, with lycopene degradation being one of the most prevalent (25, 26).

During the production of tomato juice and puree, manufacturers may add excess water or inexpensive fillers such as processing by-products and starch. From a nutritional standpoint, starch addition dilutes the natural tomato content, reducing the concentration of nutrients such as vitamins and phytochemicals. Regarding sensory attributes, excessive starch can alter the texture and mouthfeel of tomato paste, making it thicker and potentially masking quality deficiencies such as low tomato solids. Finally, the presence of undeclared starch poses a potential health risk for individuals with dietary restrictions, such as those with gluten intolerance, who may unknowingly consume a product that is unsuitable for their needs. Other potential adulterants can include sugars, acidity regulators, and even toxic synthetic dyes (27).

Having said that, the current study aimed to detect the presence of starch and assess the quality of tomato paste products in Lebanon, focusing on parameters such as total soluble solids, Bostwick consistency, viscosity, titratable acidity, color, and dry matter content, in accordance with recommended product specifications, while also evaluating labeling information.

## 2 Materials and methods

**Figure 1:**
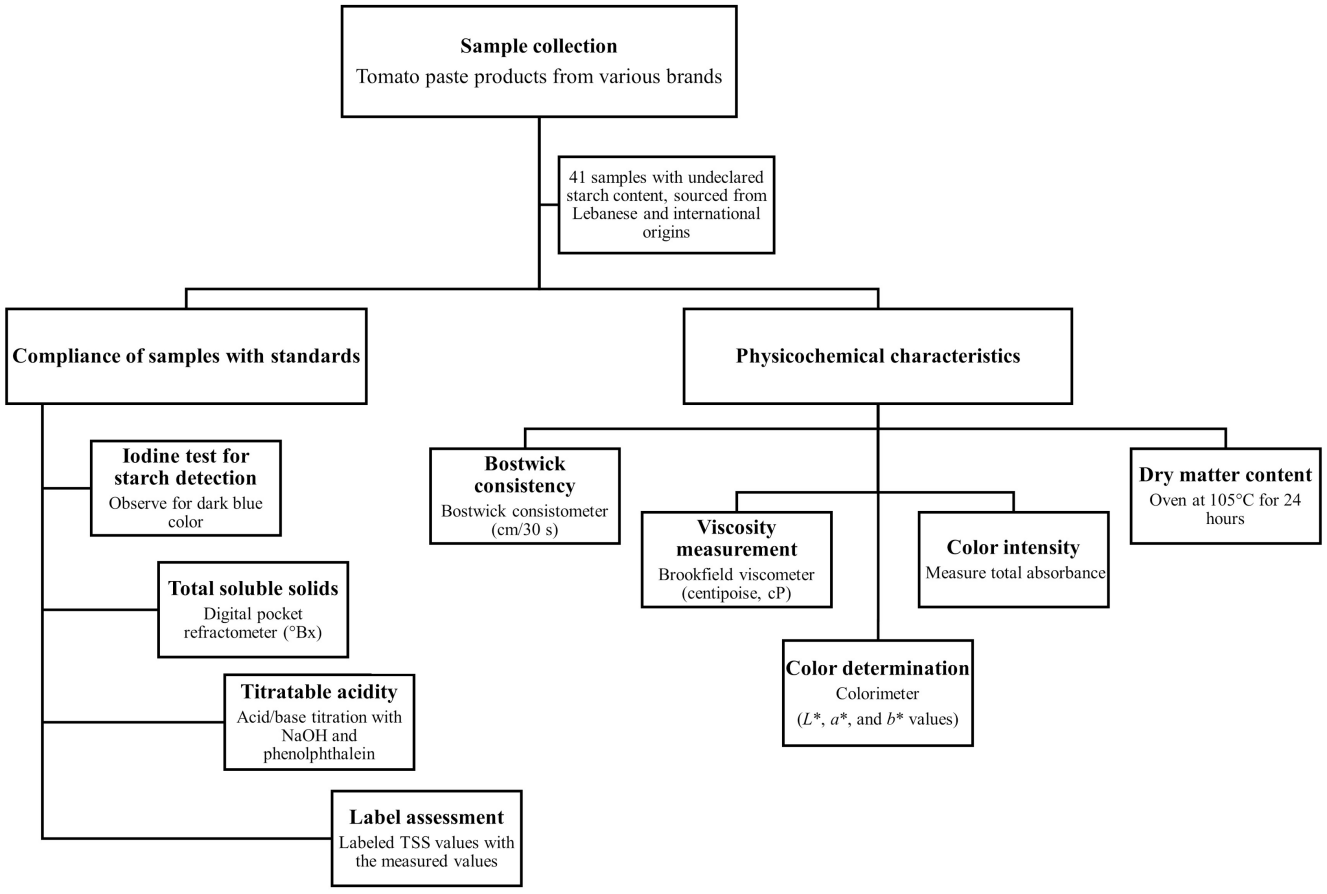
Flowchart summarizing the quality assessment of tomato paste samples.

Figure 1 displays a flowchart summarizing the quality assessment of tomato paste samples.

### 2.1 Raw materials

Tomato paste products from various brands, none of which declared starch content, were purchased in the Lebanese market, with a total of 41 items collected from different regions, including Beirut, Bekaa, and Tripoli. Out of the 41 samples, 31 samples were of Lebanese origin, while the remaining 10 were imported from Syria, Iran, Egypt, Saudi Arabia (KSA), the United Arab Emirates (UAE), Bulgaria, and China. Samples were stored in a cool, dry place, away from light, until analysis.

### 2.2 Iodine test

An iodine test was performed to detect starch in tomato paste products. First, an iodine solution consisting of 0.1% iodine (Scharlau, Barcelona, Spain) mixed with a 2% potassium iodide (Sigma-Aldrich, Steinheim, Germany) solution was prepared. Tomato paste samples were prepared by diluting one teaspoon of paste in 10 mL of distilled water. A starch-water solution was also created as a control. Next, a few drops of the iodine solution were added to each tomato paste sample in duplicate. The presence of starch was indicated by the development of a dark blue color, confirming its presence (28).

### 2.3 Total soluble solids

The total soluble solids content (TSS) was measured using a digital pocket refractometer (Sinotech, Shanghai, China). Following calibration with distilled water, the instrument provided the TSS in degrees Brix (°Bx) units, based on the refractive index of the samples. TSS reflects the soluble components in the samples, primarily indicating the sugar content in fruits and tomato products. In this context, TSS and sugar content are often expressed in °Bx, making the two terms interchangeable (29).

### 2.4 Titratable acidity

The titratable acidity (TA) was measured using an acid/base titration with 0.1 N sodium hydroxide (NaOH; Loba Chemie, Mumbai, India) and phenolphthalein indicator (1% m/v; Merck, Darmstadt, Germany) (30). The titration was performed twice, and the average value was recorded. Acidity was calculated using the formula:


$$\%\mathrm{Acidity}=\frac{\mathrm{Titer}\;\mathrm{value}\;x\;0.007009}{\mathrm{Volume}\;\mathrm{of}\;\mathrm{the}\;\mathrm{sample}}$$

Where 0.007009 is a factor for citric acid (31).

### 2.5 Bostwick consistency

Following the analysis of soluble solid content, the tomato paste samples were diluted to a concentration of 12% using distilled water for consistency testing. Each sample was placed individually into the sample chamber of the Bostwick consistometer. Upon releasing the gate fitted with a spring on one side of the chamber, the sample flowed along the sloped surface due to its weight. The distance traveled by each sample was measured after 30 s in centimeters (cm) (32, 33).

### 2.6 Viscosity

Viscosity at room temperature (25°C) was measured using a Brookfield DV-II+ Pro viscometer (Brookfield Engineering Laboratories Inc., United States) equipped with an S64 spindle operating at 100 rpm, with results reported in centipoise (cP) (34).

### 2.7 Color determination

The color of tomato paste products was evaluated using a colorimeter (BCM 200, BIOBASE, Shandong, China), configured to operate within the CIE system, which stands for the International Commission on Illumination. The instrument measured three key parameters: lightness (L*), red/green (a*), and yellow/blue (b*) (35). These values were defined as follows: L* ranges from 0 (black) to 100 (white), a* represents the red-green axis (negative values indicate green and positive values indicate red), and b* represents the blue-yellow axis (negative values indicate blue and positive values indicate yellow). For the two chromatic components (a* and b*), the value of 0 represents a neutral color.

### 2.8 Color intensity

Color intensity (CI) was assessed by measuring the absorbance of diluted samples (1:100 g/mL) at wavelengths of 420 nm (yellow), 520 nm (red), and 620 nm (blue). The CI was calculated as the total of these three absorbance measurements (36, 37). A UV–VIS spectrophotometer (GENESYS 10 UV, Thermo Electron Corporation, Waltham, MA, United States) equipped with 1 cm path length rectangular quartz cuvettes was employed for this analysis.

### 2.9 Dry matter content

The dry matter (DM) content was determined by placing samples in a well-ventilated oven at 105°C for 24 h, following the procedure described in the literature (38). The sample weights were recorded before and after drying, and the average DM content was calculated and expressed as a percentage (% w/w).

### 2.10 Labeling information assessment

Each product’s origin, price, and labeled shelf life were recorded. Additionally, a comparison was made between the labeled and tested TSS values.

### 2.11 Statistical analysis

All experiments were repeated three times to ensure the validity and reproducibility of the results. Data are expressed as mean values ± standard deviations (SDs). Statistical significance was assessed using IBM-SPSS Statistics for Windows, Version 25.0 (Released 2017, IBM Corp., New York, NY, United States). A *t*-test was applied to assess significant differences between means and paired mean differences, while the Chi-square (*χ*^2^) test was used for qualitative variables. *p*-values less than 0.05 were considered statistically significant, indicating a confidence level of over 95%.

## 3 Results and discussion

### 3.1 Compliance of tomato paste samples with standards

Table 1 presents the compliance of 41 tomato paste samples with standards for starch usage, TSS, and acidity, as defined by Libnor NL 767:2012 and Codex Stan 57–1981. Starch usage is prohibited in tomato paste products under these standards, and 37% of the samples were not compliant (Table 1). In a study by Boakye et al. (39), all tomato paste products tested in the Ghanaian market were found to contain starch. The intentional addition of this bulking agent highlights the need for stricter regulatory control to ensure transparency for consumers.

**Table 1:** Compliance of 41 tomato paste samples according to starch, total soluble solids (TSS), and titratable acidity (TA) standards by Libnor and Codex.

**Parameter**	**Standards**		**Mean ±SD** ^**a**^	**No. of samples (%)**	
	**Libnor NL 767:2012**	**Codex Stan 57-1981**		**No. compliant**	**No. non-compliant**
**Starch Usage**	Not Allowed		-	26 (63%)	15 (37%)
**TSS test**	>24%		24.21 ± 4.55%	30 (73%)	11 (27%)
**TSS label**	Range within 2% (example: 24% to 26%)		-	24 (59%)	17 (41%)
**TA**	<7%		2.19 ± 0.77%	41 (100%)	0 (0%)

^a^Mean of 41 samples ± standard deviation (SD).

In classifying tomato concentrates, the TSS is widely recognized as a key quality indicator in industrial practices. The mean TSS of the tested samples was 24.21 ± 4.55% (Table 1), with 73% meeting the requirement of at least 24% natural total soluble solids, thereby qualifying the product to be labeled as “tomato paste” (9). Regarding the TSS labeling, 59% of the samples complied with the labeled TSS, whereas 41% did not (Table 1). In comparison to other studies, the total solids in the tomato paste samples from the Kano market analyzed by Ndife et al. (31) ranged from 8.89 to 12.26%, which fell below the standard and, therefore, did not meet compliance requirements. Similarly, Joy reported a broader range of TSS values, from 15.15 to 30.99%, which included both compliant and non-compliant samples (40). Additionally, Aykas et al. (41) focused on the quality assessment of tomato paste in California and reported natural tomato soluble solids values ranging from 24.1 to 38.1 °Brix (mean: 29.4 ± 3.0 °Brix). These findings underline the variability in TSS values among tomato paste products and highlight the importance of adherence to established standards to ensure product quality and consistency. While most of the tested samples met the minimum TSS requirements, the presence of non-compliant samples emphasizes the need for stricter quality control measures in the production and labeling of tomato paste.

The TA of the tested samples reflects the concentration of organic acids, primarily citric acid, which is naturally abundant in tomatoes and plays a crucial role in shaping the characteristic taste and odor of tomato pastes while lowering the pH to ensure product safety (42). According to standards, the acceptable acidity level for tomato paste is less than 7% (9), and the mean acidity across the samples was 2.19 ± 0.77% (Table 1), with all samples (100%) complying with this requirement. The citric acid content in the final product is greatly affected by the maturity of the tomatoes, as well as handling and processing conditions, due to its susceptibility to degradation. To address this, citric acid is often added as an acidity regulator to maintain the desired pH levels, which should be below 4.6 according to Libnor and Codex Alimentarius standards. In comparison, the acidity of tomato paste samples in a study by Ndife et al. (31) ranged from 0.36 to 0.49%, remaining well within the maximum limit of 7% recommended by the Codex Alimentarius. Similarly, Aykas et al. (41) reported a TA range of 1.0–2.4% (1.6 ± 0.2) when assessing the quality of various tomato paste samples using portable mid-infrared spectroscopy. Devseren et al. (43) also documented a TA range of 0.727–2.318% in their investigation of tomato pastes processed under varying temperatures and processing times. These findings align closely with the mean acidity level reported in the current study (Table 1).

### 3.2 Descriptive values of tomato paste samples

The descriptive values of 41 tomato paste samples are provided in Table 2. The analysis of the samples revealed that the DM ranged from 12.77 to 29.11%, with a mean value of 22.64 ± 3.94% (Table 2). In comparison, Ndife et al. (31) reported moisture content values ranging from 89.27 to 91.11%, corresponding to dry matter values of 8.89 to 10.73%. Similarly, Eke-Ejiofor (40) reported moisture content values between 69.00 and 84.85%, which translate to dry matter values ranging from 15.15 to 31.00%. Abdullahi et al. (44) reported moisture content values of 71.80 to 72.40%, corresponding to dry matter values of 27.60 to 28.20%. Additionally, Sobowale found total solids ranging from 54.9 to 68.90%, corresponding to dry matter values of 31.10 to 45.10%. Our study’s broader range of DM values (12.77 to 29.11%) suggests a greater variability in solid content, with some samples exhibiting relatively lower dry matter than those reported in other studies.

**Table 2:** Descriptive values of 41 tomato paste samples.

**Parameter**	**Minimum**	**Maximum**	**Mean ± SD** ^**a**^
DM (%)	12.77	29.11	22.64 ± 3.94
Bostwick (cm/30 s)	0.10	5.25	1.54 ± 0.96
Viscosity (cP)	3479	4167	3979 ± 117
L*	5.55	30.28	12.38 ± 6.49
a*	4.32	36.26	11.90 ± 5.78
b*	–6.02	17.91	1.73 ± 3.36
a*/b*	–28.15	29.06	5.28 ± 12.07
Color Intensity	0.87	3.07	2.03 ± 0.55

^a^ Mean of 41 samples ± standard deviation (SD). DM, dry matter.

One of the commonly employed standard methods by industries to evaluate tomato products is the Bostwick consistency test (22). This test measures the flow of a specific sample volume (45, 46), providing information about the product’s consistency and shear-thinning properties (47). In this study, Bostwick consistency values ranged from 0.10 to 5.25 cm/30 s, with a mean value of 1.54 ± 0.96 cm/30 s” (Table 2). These values are comparable to those reported by Aykas et al. (41), who observed a broader range of 0.8 to 11.9 cm/30 s, with a mean of 3.6 ± 1.9  cm/30 s, indicating a higher average consistency compared to the samples analyzed in this study. Vitalis et al. (20) reported a Bostwick consistency of 5.83 ± 0.57 cm/30 s for authentic tomato paste samples, which is notably higher than the mean value observed in this study (1.54 ± 0.96 cm/30 s). Munhoz and Schmidt (48) investigated the impact of temperature and prolonged heating on consistency using the Bostwick device. They found no significant differences in consistency measurements at 60°C (4.0 ± 0.1 cm/30 s) and 70°C (4.1 ± 0.1 cm/30 s). However, at 80°C, consistency degradation became apparent only after 6 h of heating, with values stabilizing between 4.3 and 4.5 cm/30 s after extended heating for up to 48 h (48). The variations in Bostwick consistency values reported across different studies emphasize the influence of factors such as processing conditions, raw material characteristics, and product formulation on the rheological properties of tomato products.

Viscosity is a crucial technological parameter affected by the levels of proteins, pectins, polysaccharides, and alcohol-insoluble compounds (49). Similarly, Sobowale et al. (50) highlighted that the viscosity of tomato products is influenced by their fiber, protein, fat, and total solid content. In the present study, viscosity ranged between 3,479 cP and 4,167 cP, with a mean value of 3,979 ± 117 cP (Table 2). In contrast, Hassan et al. (51) reported much lower viscosity values, ranging from 370 cP to 489.7 cP, in their evaluation of the effects of pre-heating and concentration temperatures on tomato paste quality. Hassan et al. (51) noted that breaking temperatures (60°C, 70°C, and 90°C) and concentration temperatures (80°C and 90°C) significantly impacted viscosity, suggesting that processing temperatures play a critical role in determining the rheological properties of tomato paste (52). Additionally, the inactivation of pectolytic enzymes by heat was found to contribute to higher serum and efflux viscosity values by stabilizing pectin structures (35, 53).

The color parameters of the tomato paste samples exhibited variation. L* values ranged from 5.55 to 30.28, with a mean of 12.38 ± 6.49, indicating that the samples were generally darker in appearance. The a* ranged from 4.32 to 36.26, with a mean of 11.90 ± 5.78, suggesting a reddish tone across most samples. The b* ranged from −6.02 to 17.91, with a mean of 1.73 ± 3.36, implying a yellowish tendency. The a*/b* ratio, an important indicator of color quality, showed considerable variability, with values ranging from −28.15 to 29.06 and a mean of 5.28 ± 12.07 (Table 2). This wide range indicates that not all samples met the color quality specifications. As per U.S. and Canadian standards, the color of tomato paste is a critical quality parameter, and the a*/b* ratio is commonly used as a scale for product acceptability (43). According to Turkish and Egyptian standards, the a*/b* ratio should not be less than 1.8, as values below this threshold indicate lower quality products (43, 54). Although the mean ratio suggests that most samples met the minimum standard, the variability highlights inconsistencies in quality, with several samples failing to meet the specified standards.

The CI values in this study ranged from 0.87 to 3.07, with a mean of 2.03 ± 0.55 (Table 2). The red color of tomatoes is primarily due to carotenoids, with lycopene being the predominant pigment, accounting for about 83% of the total carotenoids, while *β*-carotene makes up around 3–7% (55–57). The concentration of these carotenoids can vary based on the tomato variety and growing conditions (55). Additionally, brown pigments in processed tomato products may form due to Maillard reactions or caramelization of sugars. These processes are influenced by factors such as the sugar and amino acid content, pH, and processing conditions, including time and temperature (22). This variability in the factors influencing color could help explain the range of color intensity observed in the tomato paste samples in this study.

### 3.3 Starch conformity and physicochemical characteristics of tomato paste

The correlation analysis between starch conformity and the physicochemical characteristics of tomato paste samples is summarized in Table 3. Compliant samples (*n* = 26) exhibited significantly higher TSS values (26.06 ± 2.89%) compared to non-compliant samples (21.23 ± 5.18%, *p* < 0.01). Acidity was also notably higher in compliant samples (2.61 ± 0.56%) than in non-compliant ones (1.47 ± 0.50%, *p* < 0.001). Similarly, dry matter (DM) content was significantly greater in compliant samples (24.07 ± 2.85%) versus non-compliant samples (20.16 ± 4.41%, *p* < 0.01). Additionally, CI was significantly higher in compliant samples (2.34 ± 0.41) compared to non-compliant samples (1.49 ± 0.32, *p* < 0.001). However, no significant differences were observed between compliant and non-compliant samples for Bostwick consistency (1.60 ± 0.62 cm/30 s vs. 1.44 ± 1.39 cm/30 s), viscosity (3,967 ± 141 cP vs. 3,998 ± 54 cP), color parameters (L* (11.87 ± 6.37 vs. 13.27 ± 6.81), a* (11.54 ± 6.66 vs. 12.52 ± 3.95), b* (2.11 ± 3.82 vs. 1.08 ± 2.34), a*/b* (3.07 ± 12.09 vs. 9.11 ± 11.42)), shelf life (2.04 ± 0.37 years vs. 1.92 ± 0.28 years), or price ($1.16 ± 0.78 vs. $0.84 ± 0.42) (Table 3), indicating no significant relationship with starch conformity (*p* > 0.05). These results suggest that starch conformity is associated with specific physicochemical characteristics, particularly TSS, TA, DM, and CI, but not with the tomato paste’s consistency, viscosity, color, shelf life, or price.

**Table 3:** Correlation between starch conformity and physicochemical characteristics of tomato paste.

**Parameter**	**Starch conformity (*n* = 41)**		
	**No. compliant (*n* = 26)**	**No. non-compliant (*n* = 15)**	**Significance**
TSS (%)	26.06 ± 2.89	21.23 ± 5.18	**
TA (%)	2.61 ± 0.56	1.47 ± 0.50	***
DM (%)	24.07 ± 2.85	20.16 ± 4.41	**
Bostwick (cm/30 s)	1.60 ± 0.62	1.44 ± 1.39	NS
Viscosity (cP)	3967 ± 141	3998 ± 54	NS
L*	11.87 ± 6.37	13.27 ± 6.81	NS
a*	11.54 ± 6.66	12.52 ± 3.95	NS
b*	2.11 ± 3.82	1.08 ± 2.34	NS
a*/b*	3.07 ± 12.09	9.11 ± 11.42	NS
CI	2.34 ± 0.41	1.49 ± 0.32	***
Shelf life (years)	2.04 ± 0.37	1.92 ± 0.28	NS
Price ($)	1.16 ± 0.78	0.84 ± 0.42	NS

Data are presented as mean ±SD. *n* is the sample size. Not Significant (NS): *p* > 0.05, *: *p* < 0.05, **: *p* < 0.01, ***: *p* < 0.001, where ∗, ∗∗, and ∗∗∗ denote levels of significance difference. TSS, total soluble solids; TA, titratable acidity; DM, dry matter; CI, color intensity.

Shatta (58) demonstrated that as the TSS in tomato paste from Egypt increased from 4.48 to 35.88%, the moisture content significantly decreased from 94.17 to 63.13%, while TA increased notably from 0.45 to 3.00%. These findings align with our results, where compliant tomato paste samples had higher TSS (26.06 ± 2.89%), greater acidity (2.61 ± 0.56%), and higher dry matter (24.07 ± 2.85%) compared to non-compliant samples. The presence of starch in non-compliant samples (*n* = 15) likely contributed to their lower mean TSS (21.23 ± 5.18%), which fell below the required 24% standard. TSS reflects the concentration of natural sugars, such as sucrose and fructose, in tomato paste, with higher TSS indicating a sweeter, more concentrated product (39). Our findings further support this, as samples containing starch had reduced TSS levels, highlighting the negative impact of starch addition on TSS compliance.

The presence of starch does not appear to significantly impact titratable acidity, as the non-compliant samples still exhibit TA levels below the acceptable acidity threshold for tomato paste, which is less than 7% (9).

Total solids are a key indicator of moisture content, with higher total solids reflecting better-quality tomato paste, as defined by food standards (50). Lower dry matter content (20.16 ± 4.41), associated with higher moisture levels, suggests that water may have been added to the non-compliant samples. This, coupled with the presence of starch, could explain the reduced dry matter content observed in these samples, pointing to potential adulteration aimed at enhancing perceived quality.

The CI was significantly lower in non-compliant samples (1.49 ± 0.32) compared to compliant ones (2.34 ± 0.41). This reduction suggests that starch addition dilutes key pigments, such as lycopene and *β*-carotene, which are primarily responsible for the vibrant red color of tomato paste (55, 57).

### 3.4 Parameters with country of origin of tomato paste

Table 4 presents the correlation between various parameters of tomato paste and its country of origin, with a comparison between local and imported products. For TSS, the average values for local and imported tomato paste were 24.10 ± 4.96% and 24.50 ± 3.24%, respectively, with no significant difference (*p* = 0.816). TA levels were also similar, with local samples having 2.14 ± 0.83% and imported samples 2.36 ± 0.59%, with insignificant differences (*p* = 0.436). DM content for both groups was comparable (22.65 ± 4.20% for local and 22.61 ± 3.20% for imported), and the difference was not significant (*p* = 0.978). Other parameters, including Bostwick consistency (1.53 ± 1.01 cm/30 s for local and 1.58 ± 0.81 cm/30 s for imported, *p* = 0.901) and viscosity (3,976 ± 135 cP for local and 3,988 ± 20 cP for imported, *p* = 0.775), also did not differ significantly. Colorimetric attributes such as lightness (L*), redness (a*), yellowness (b*), and the a*/b* ratio were consistent between local and imported products, with *p*-values of 0.800, 0.515, 0.430, and 0.486, respectively. CI was slightly higher in imported samples (2.21 ± 0.42) compared to local samples (1.97 ± 0.58), but the difference was not statistically significant (*p* = 0.165). Lastly, shelf life and price also showed no significant variation. Local products had an average price of 1.07 ± 0.75$, slightly higher than the imported average of 0.97 ± 0.43$ (*p* = 0.705). Overall, these findings indicate that local and imported tomato paste products are highly similar in terms of their physicochemical properties and quality indicators.

**Table 4:** Correlation between parameters and tomato paste country of origin.

**Parameter**	**Country of Origin**		
	**Local**	**Imported**	**Significance**
	**(*n* = 31)**	**(*n* = 10)**	
TSS (%)	24.10 ± 4.96	24.50 ± 3.24	NS
TA (%)	2.14 ± 0.83	2.36 ± 0.59	NS
DM (%)	22.65 ± 4.20	22.61 ± 3.20	NS
Bostwick (cm/30 s)	1.53 ± 1.01	1.58 ± 0.81	NS
Viscosity (cP)	3976 ± 135	3988 ± 20	NS
L*	12.53 ± 6.91	11.92 ± 5.26	NS
a*	12.24 ± 6.21	10.85 ± 4.26	NS
b*	1.50 ± 3.74	2.48 ± 1.66	NS
a*/b*	4.52 ± 13.24	7.63 ± 7.42	NS
CI	1.97 ± 0.58	2.21 ± 0.42	NS
Shelf life (years)	2.00 ± 0.45	2.00 ± 0.25	-
Price ($)	1.07 ± 0.75	0.97 ± 0.43	NS

Data are presented as mean ±SD. *n* is the sample size. Not Significant (NS): *p*>0.05. TSS, total soluble solids; TA, titratable acidity; DM, dry matter; CI, color intensity.

### 3.5 Compliance of tomato paste origin with starch usage and TSS

**Figure 2:**
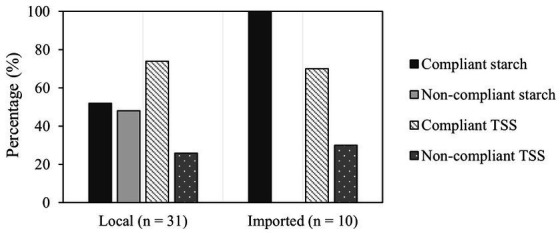
Local (*n* = 31) and imported (*n* = 10) tomato paste samples tested for compliance with starch and total soluble solids (TSS).

Figure 2 illustrates the compliance of local and imported tomato paste samples with starch usage and TSS levels. Among local samples (*n* = 31), 52% adhered to the starch usage standard, while 74% met the required TSS level. On the other hand, all imported samples from Syria (*n* = 4), Iran (*n* = 1), Egypt (*n* = 1), KSA (*n* = 1), UAE (*n* = 1), Bulgaria (*n* = 1), and China (*n* = 1) complied with starch usage. Specifically, 70% of imported samples from Syria, Iran, KSA, and Bulgaria complied with TSS standards, except for those from Egypt, UAE, and China.

To the best of our knowledge, no previous study has compared the compliance of starch usage in tomato paste products of Lebanese origin to those from other countries in the Lebanese market. Importantly, our findings revealed that 48% of the Lebanese tomato paste samples (*n* = 31) did not comply with the starch usage standard, meaning that nearly half of the products (*n* = 15) failed to meet the required guidelines. This highlights a significant gap in the quality control of Lebanese tomato paste, emphasizing the need for stricter regulations, transparent labeling, and a more rigorous oversight. To address these compliance issues and effectively combat food fraud, Lebanese regulatory authorities should prioritize the collection and testing of tomato paste samples for adulteration. Inspectors should be empowered to conduct on-the-spot tests, such as the iodine test and TSS measurement, to quickly identify adulterated products. A clear enforcement protocol should be established, starting with a verbal warning for violations, followed by penalties and sanctions if issues are not corrected within a specified timeframe. Persistent non-compliance could result in product recalls or facility closures. These actions will not only strengthen compliance but also protect consumers and ensure a fair market.

## 4 Conclusion

This study evaluated the quality and potential adulteration of tomato paste products available in the Lebanese market, focusing on starch usage and various physicochemical parameters. The analysis of 41 tomato paste samples revealed that 37% were non-compliant with starch usage standards, highlighting a need for stricter enforcement of existing regulations. While 73% of the samples met the required TSS of 24, 41% did not match their labeled TSS. Nevertheless, all samples adhered to the acidity standards of less than 7%, confirming their safety in terms of pH levels. A comparison between compliant and non-compliant samples revealed that compliant products had significantly higher values for TSS, TA, DM, and CI. These parameters serve as quality indicators and emphasize the impact of adulteration on the overall product composition. Notably, the study found no significant differences in physicochemical properties, color, shelf life, or price between national and international tomato paste products, suggesting similar overall quality across samples. However, 48% of local samples were non-compliant with starch usage standards, while imported products adhered to these standards. While this study provides important insights into the adulteration of tomato paste in the Lebanese market, there are certain limitations to be acknowledged. The sample size of 41 products and the focus on a single region may limit the generalizability of the findings to other markets. Additionally, starch detection was based on qualitative analysis, which, although effective for identifying adulteration, lacks the precision of quantitative methods. Future research should aim to include larger sample sizes, broader geographic coverage, and quantitative techniques to further enhance the reliability and validity of the results.

## Data Availability

The original contributions presented in the study are included in the article/supplementary material, further inquiries can be directed to the corresponding author.
